# Biochemical Endocrine and Skeletal Findings in Adults with Fabry Disease: A Descriptive Single-Center Study

**DOI:** 10.3390/biom16101395

**Published:** 2026-09-24

**Authors:** Sadettin Ozturk, Ilkcan Cerci Kocar

**Affiliations:** 1 Department of Endocrinology and Metabolism, Gaziantep City Hospital, 27100 Gaziantep, Türkiye; 2 Department of Endocrinology and Metabolism, Mersin City Training and Research Hospital, 33343 Mersin, Türkiye; dr.ilkcan_33@hotmail.com

**Keywords:** Fabry disease, endocrine abnormalities, dysglycemia, vitamin D deficiency, bone mineral density, Lyso-Gb3, multiorgan involvement, enzyme replacement therapy

## Abstract

**Background:** Endocrine and skeletal findings have previously been reported in Fabry disease (FD), but their interpretation alongside Fabry-specific biochemical markers and multiorgan disease burden remains uncertain. This study descriptively characterized biochemical endocrine findings, vitamin D status, and age-appropriate DXA measurements in adults with FD receiving enzyme replacement therapy (ERT). **Methods:** This retrospective, single-center study screened 29 patients evaluated between January 2020 and December 2025, of whom 21 adults with genetically and/or enzymatically confirmed FD were included. Biochemical thyroid findings and HbA1c-defined dysglycemia were summarized separately as descriptive measures; no prespecified primary endpoint, composite endocrine outcome, or confirmatory outcome hierarchy was defined. A post hoc exploratory comparison was performed between patients with HbA1c-defined dysglycemia (HbA1c ≥ 5.7%; *n* = 7) and those without HbA1c-defined dysglycemia (HbA1c < 5.7%; *n* = 14). Vitamin D status and DXA findings were evaluated using age-, sex-, and menopausal-status-appropriate criteria. Exploratory analyses incorporated effect sizes, bootstrap confidence intervals, permutation tests, and Benjamini–Hochberg correction. **Results:** The mean age was 40.7 ± 14.5 years, and nine patients (42.9%) were male. The cohort was genetically concentrated, with c.851T>C (p.M284T) identified in 12/21 patients (57.1%). An elevated-TSH/non-elevated-FT4 biochemical pattern was observed in 2/21 patients (9.5%), and HbA1c-defined dysglycemia in 7/21 (33.3%). Vitamin D concentrations were <20 ng/mL in 16/21 patients (76.2%). DXA data were available for 20 patients. Among four postmenopausal women and four men aged ≥50 years, 5/8 (62.5%) had a minimum T-score within the osteoporosis range. Among seven premenopausal women and five men aged <50 years, 7/12 (58.3%) had a minimum Z-score ≤ −2.0, indicating BMD below the expected range for age. None of the 14 post hoc subgroup comparisons or 48 exploratory correlations remained significant after Benjamini–Hochberg correction. **Conclusions:** Thyroid, glycemic, vitamin D, and age-appropriate DXA measurements were descriptively characterized in this small, ERT-treated FD cohort. In the absence of an age-, sex-, and clinically matched comparator, the observed distributions cannot be interpreted as abnormalities attributable specifically to FD or as occurring more frequently than in an appropriate reference population. Moreover, no subgroup comparison or correlation remained significant after multiplicity correction. The data therefore do not demonstrate independent endocrine or skeletal relationships with Fabry disease burden and should be considered cohort-specific, descriptive, and hypothesis-generating.

## 1. Introduction

Fabry disease (FD; OMIM 301500) is a rare, progressive, X-linked lysosomal storage disorder caused by pathogenic variants in the *GLA* gene, resulting in deficient α-galactosidase A (α-Gal A) activity. The enzymatic defect leads to the progressive accumulation of globotriaosylceramide (Gb3), globotriaosylsphingosine (Lyso-Gb3), and related glycosphingolipids in plasma and within the lysosomes of endothelial cells, cardiomyocytes, renal podocytes, neurons, and other cell types [1,2]. In addition to substrate accumulation, impaired autophagy, oxidative stress, chronic inflammation, endothelial dysfunction, and progressive fibrosis contribute to cellular injury and irreversible organ damage [3,4,5,6].

The clinical presentation of FD is highly heterogeneous and is influenced by sex, age, residual α-Gal A activity, the underlying *GLA* variant, and X-chromosome inactivation patterns in women [7,8]. Patients with the classic phenotype may develop acroparesthesia, pain crises, hypohidrosis, angiokeratomas, cornea verticillata, gastrointestinal symptoms, tinnitus, and exercise intolerance during childhood or adolescence. Progressive renal, cardiac, and cerebrovascular complications generally become more prominent in adulthood. Renal involvement may manifest as albuminuria, proteinuria, declining glomerular filtration rate, and kidney failure, whereas cardiac disease includes left ventricular hypertrophy, myocardial fibrosis, conduction abnormalities, arrhythmias, and heart failure. Neurological involvement ranges from small-fiber neuropathy and autonomic dysfunction to transient ischemic attacks and stroke [9,10,11,12]. Gastrointestinal, auditory, vestibular, dermatological, and ocular manifestations further increase the overall disease burden. Accordingly, current recommendations emphasize individualized and multidisciplinary surveillance [8,13].

Endocrine involvement in FD has been evaluated in several previous studies. Reported findings include antibody-negative subclinical hypothyroidism, reproductive and gonadal abnormalities, impaired semen parameters, vitamin D deficiency, and alterations in metabolic and hypothalamic–pituitary function [14,15,16,17]. However, the results have varied across cohorts, and a prospective reference-center study found no major systematic dysfunction of the adrenal, growth hormone, or sex-hormone axes despite identifying thyroid abnormalities, vitamin D deficiency, and other endocrine conditions [16]. Thus, the unresolved issue is not whether endocrine abnormalities can occur in patients with FD, but how frequently they are observed in specific clinical cohorts and how they should be interpreted in relation to age, conventional comorbidities, Fabry-specific biochemical burden, treatment exposure, and established organ involvement. Available studies have generally evaluated individual endocrine axes or reported their frequencies without integrating these findings with renal parameters, validated disease-severity scores, and a broad assessment of multiorgan involvement within the same treated cohort [14,15,16,17].

Reduced BMD is an established, although incompletely characterized, finding in FD. Previous cross-sectional studies have documented osteopenia, osteoporosis, and lower-than-expected BMD, while more recent DXA-based and longitudinal analyses have examined associations with Lyso-Gb3, renal function, conventional skeletal risk factors, and Fabry-specific treatment [18,19,20]. Collectively, these studies indicate that low BMD is not a previously unrecognized manifestation of FD. Nevertheless, its clinical interpretation remains challenging because BMD is strongly influenced by age, sex, menopausal status, body composition, physical activity, renal dysfunction, vitamin D status, hypogonadism, and medication exposure. Moreover, available evidence has not established whether the observed skeletal abnormalities are directly attributable to FD or primarily reflect conventional and disease-related comorbid factors. The present study therefore did not seek to re-establish an increased prevalence of low BMD or determine the effect of ERT. Instead, it descriptively evaluated age- and sex-appropriate DXA findings and explored whether skeletal measurements co-varied with contemporaneous biochemical and clinical indicators of Fabry disease burden.

Lyso-Gb3, α-Gal A activity, Fabry-specific treatment exposure, and validated disease-severity scores may provide clinical context when characterizing treated FD cohorts [21,22,23,24,25]. In the present study, however, these variables were included only as contextual measures in post hoc exploratory analyses and were not evaluated as prespecified endpoints, validated predictors, or measures of treatment response.

Against this background, the objective of the present study was to descriptively characterize the observed frequencies and distributions of biochemical thyroid and glycemic findings, vitamin D status, and age-, sex-, and menopausal-status-appropriate DXA findings in adults with confirmed FD receiving ERT. Additional post hoc analyses comprised an HbA1c-defined dysglycemia subgroup comparison across selected demographic, treatment-related, and disease-burden variables, together with correlations of selected endocrine and skeletal measurements with disease duration, Lyso-Gb3, MSSI, DS3, eGFR, and proteinuria. These analyses were intended solely to generate hypotheses regarding the clinical context in which these measurements coexist rather than to establish a distinct endocrine phenotype, estimate excess prevalence attributable to FD, infer causal relationships, assess biomarker performance, or determine the effect of treatment.

## 2. Materials and Methods

### 2.1. Study Design and Participants

This retrospective, single-center observational study was conducted at the Department of Endocrinology and Metabolism, Gaziantep City Hospital, Türkiye. The institutional records and electronic medical files of all patients with a documented or suspected diagnosis of Fabry disease who were evaluated or regularly followed at the institution between 1 January 2020, and 31 December 2025, were screened for eligibility. Potentially eligible patients were identified through the hospital electronic medical record system and the departmental Fabry disease follow-up list.

The study period refers to the dates during which routine clinical care was provided and the corresponding medical records were generated, rather than to the period of research data collection. On 21 January 2026, the Gaziantep City Hospital Non-Interventional Clinical Research Ethics Committee approved the protocol, including retrospective access to and analysis of records generated between 1 January 2020, and 31 December 2025 (Approval No. 419/2026). Research-specific record review and data extraction commenced only after ethics approval. After approval and before their records were included in the research dataset, all participants provided written informed consent specifically authorizing the use of information obtained during routine clinical follow-up for the purposes of this study. This research consent was distinct from consent for routine clinical care or diagnostic procedures, and no additional intervention, examination, or blood sampling was performed for the study.

Patients were eligible if they were aged 18 years or older; had Fabry disease confirmed by molecular genetic testing and/or demonstration of deficient α-galactosidase A activity; had regular outpatient follow-up at the institution; and had sufficient core clinical and laboratory information for the descriptive assessment. Patients were excluded if they were younger than 18 years; did not have genetically or enzymatically confirmed Fabry disease; did not have regular clinical follow-up; were receiving an investigational Fabry-specific treatment other than standard ERT; had an active infection or acute inflammatory condition at the index assessment; had advanced active malignancy; were pregnant or breastfeeding; or had insufficient or inaccessible core medical records.

All potentially eligible records identified during the study period were evaluated. Each excluded patient was assigned a single primary exclusion reason according to a predefined hierarchical order to prevent double counting. The absence of DXA measurements or selected gonadal, adrenal, or pituitary measurements did not result in exclusion when the required core clinical and biochemical information was available. Variables with incomplete measurements were evaluated using available-case denominators without imputation.

### 2.2. Data Collection and Treatment

For this retrospective cross-sectional analysis, an index date was defined separately for each patient as the date of the most recent routine Fabry follow-up visit during the study period at which the core biochemical measures used to characterize thyroid and glycemic status—TSH, free thyroxine, and HbA1c—were obtained concurrently. The index date was selected without reference to the values or direction of these measurements. Age, disease duration, and Fabry-specific treatment duration were calculated as of this date. Other routine endocrine, metabolic, and renal laboratory measurements were selected from the index date or, when unavailable on that date, from the measurement closest to the index date within ±30 days. Urinary protein or albumin measurements, serum Lyso-Gb3 concentrations, and MSSI and DS3 assessments were selected from the observations closest to the index date within ±90 days. The DXA examination closest to the index date within ±180 days was used. When two eligible measurements were equally distant from the index date, the earlier measurement was selected. Measurements outside the prespecified windows were treated as unavailable, and no imputation or last-observation-carried-forward procedure was applied. Chronic renal, cardiac, neurological, and other organ involvement was determined from diagnoses and investigations documented on or before the index date; events first documented after the index date were not included in the cross-sectional assessment.

The following variables were extracted from the medical records: age, sex, age at diagnosis, disease duration, family history of Fabry disease, *GLA* variant, ERT status, type of ERT, and treatment duration. Disease duration was calculated as the interval between the documented date of Fabry disease diagnosis and the patient-specific index date. For treated patients, Fabry-specific treatment duration was calculated from treatment initiation to the index date and expressed in years.

Patients receiving ERT were treated according to routine clinical practice with either agalsidase alfa (Replagal^®^, Takeda Pharmaceutical Company, Tokyo, Japan) at a dose of 0.2 mg/kg every two weeks or agalsidase beta (Fabrazyme^®^, Sanofi, Paris, France) at a dose of 1 mg/kg every two weeks. Information regarding treatment interruptions, switching between ERT products, and treatment-related adverse events was recorded when available.

Serum Lyso-Gb3 concentration was recorded as a Fabry disease-related biochemical measure. α-Gal A activity was also recorded when available. Serum Lyso-Gb3 concentrations were quantified using a validated ultra-high-performance liquid chromatography–tandem mass spectrometry (UHPLC–MS/MS) method at the Central Biochemistry Laboratory of Gaziantep City Hospital. Quantitative analysis was performed using a Sciex 4500 QTRAP triple-quadrupole mass spectrometer (Sciex, Concord, ON, Canada) coupled to a Nexera X2 UHPLC system (Shimadzu Corporation, Kyoto, Japan). Chromatographic separation was achieved using a Kinetex C18 column (Phenomenex, Torrance, CA, USA), and Lyso-Gb3 was detected in multiple-reaction monitoring positive-ionization mode using isotope-labeled Lyso-Gb3-D7 (Merck KGaA, Darmstadt, Germany) as the internal standard. The analytical measuring range was 0.25–100 ng/mL, and the lower limit of quantification was 0.25 ng/mL. The intra-assay and inter-assay coefficients of variation were both <10% [26]. For the cross-sectional analysis, the serum Lyso-Gb3 measurement closest to the patient-specific index date within ±90 days was selected. Samples were collected during routine outpatient follow-up, and sampling was not standardized in relation to ERT administration. If two eligible measurements were equally distant from the index date, the earlier measurement was used. Measurements outside this window were considered unavailable. Samples were obtained during routine outpatient follow-up, and their timing was not standardized relative to ERT administration. When more than one eligible measurement was available within the predefined window, the measurement temporally closest to the index assessment was used; if two measurements were equally distant, the earlier measurement was selected.

Routine hematological and biochemical variables included hemoglobin, hematocrit, white blood cell, neutrophil, lymphocyte and platelet counts, total protein, albumin, fasting plasma glucose, HbA1c, total cholesterol, low-density lipoprotein cholesterol, high-density lipoprotein cholesterol, and triglycerides.

Endocrine assessment included serum thyroid-stimulating hormone, free thyroxine, free triiodothyronine, follicle-stimulating hormone, luteinizing hormone, estradiol, total testosterone, anti-Müllerian hormone, inhibin B, adrenocorticotropic hormone, cortisol, prolactin, growth hormone, and insulin-like growth factor 1, where available. Menopausal status, menstrual irregularity, infertility history, clinical evidence of hypogonadism, thyroid medication use, and other hormone replacement treatments were also recorded.

Glucose metabolism was evaluated using fasting plasma glucose and HbA1c. Fasting insulin and the homeostatic model assessment of insulin resistance were evaluated when available. The homeostatic model assessment of insulin resistance was calculated as follows:

$$\mathrm{HOMA}\text{-}\mathrm{IR}=\frac{\mathrm{fasting}\;\mathrm{insulin}\;\left(\mu U/\mathrm{mL}\right)\times \mathrm{fasting}\;\mathrm{glucose}\;\left(\mathrm{mg}/\mathrm{dL}\right)}{405}$$

Bone and mineral metabolism was evaluated using serum calcium, phosphorus, magnesium, 25-hydroxyvitamin D, and parathyroid hormone concentrations. All laboratory results were interpreted according to the sex-, age-, and assay-specific reference intervals of the hospital laboratory.

### 2.3. Endocrine and Bone Health Assessment

Endocrine findings were classified at two distinct levels. A clinically established endocrine disorder required a documented clinical diagnosis supported by appropriate confirmatory biochemical or dynamic testing and/or ongoing disease-specific treatment. In contrast, isolated abnormalities identified from the available laboratory measurements were classified as biochemical endocrine findings and were not automatically considered equivalent to clinically confirmed endocrine disease.

Biochemical thyroid dysfunction was operationally defined as a TSH concentration > 4.0 mIU/L accompanied by a non-elevated FT4 concentration. Because thyroid autoantibodies, thyroid ultrasonography, repeat thyroid function testing, and complete treatment histories were not uniformly available, this finding was described as a biochemical elevated-TSH/non-elevated-FT4 pattern rather than confirmed primary hypothyroidism. Adrenal insufficiency and growth hormone abnormalities were accepted only when supported by an established clinical diagnosis, appropriate dynamic testing, or a consistent hormonal pattern. Isolated random cortisol or growth hormone measurements were not considered diagnostic.

Glucose metabolism was evaluated using HbA1c and fasting plasma glucose where available. HbA1c values of 5.7–6.4% were classified as being within the prediabetes range, while values ≥ 6.5% were classified as being within the diabetes range. In the absence of repeat confirmatory testing, compatible symptoms, or an established clinical diagnosis, these categories were considered biochemical dysglycemia rather than confirmed prediabetes or diabetes mellitus.

The elevated-TSH/non-elevated-FT4 pattern and HbA1c-defined dysglycemia were summarized separately as descriptive biochemical findings. For descriptive reporting, serum 25-hydroxyvitamin D concentrations were categorized as <20 or ≥20 ng/mL. Gonadal measurements and DXA findings were evaluated separately. No composite endocrine outcome was defined.

DXA examinations were performed by trained technicians using a single Lunar Prodigy Advance densitometer (GE Healthcare, Madison, WI, USA) equipped with enCORE software version 18.0. All examinations were performed on the same instrument. The densitometer underwent daily calibration using the manufacturer-supplied quality-assurance phantom, and routine quality-control procedures were performed in accordance with the manufacturer’s recommendations. No major hardware or software changes occurred during the study period. Areal BMD was measured at the lumbar spine (L1–L4), femoral neck, and total hip and was expressed as g/cm^2^. Scans were analyzed according to standard positioning and acquisition procedures, and vertebrae affected by structural abnormalities or technical artifacts were excluded in accordance with ISCD recommendations. Patient-level classification was based on the lowest valid score obtained at the lumbar spine, femoral neck, or total hip. T-scores were used only for postmenopausal women and men aged ≥50 years and were categorized as normal (≥−1.0), low bone mass (between −1.0 and −2.5), or within the osteoporosis range (≤−2.5). In premenopausal women and men aged <50 years, Z-scores were used; a Z-score ≤ −2.0 was classified as BMD below the expected range for age. A Z-score ≤ −2.0 was not considered sufficient by itself to establish a diagnosis of osteoporosis [27]. Site-specific T-score values were summarized only among postmenopausal women and men aged ≥50 years, whereas site-specific Z-score values were summarized only among premenopausal women and men aged <50 years. Areal BMD was summarized among all patients with a valid measurement at the corresponding skeletal site, and the number of contributing patients was reported for each estimate.

### 2.4. Multiorgan Involvement and Disease Severity Assessment

Renal assessment included serum urea, creatinine, estimated glomerular filtration rate, uric acid, proteinuria, albuminuria, hematuria, renal ultrasonography findings, and the presence of chronic kidney disease or kidney failure. Estimated glomerular filtration rate values were extracted from the laboratory records. Proteinuria and albuminuria were evaluated using available 24 h urine measurements or spot urine protein- or albumin-to-creatinine ratios. Renal involvement was considered present when at least one clinically relevant Fabry-associated renal abnormality, including persistent albuminuria or proteinuria, reduced estimated glomerular filtration rate, documented Fabry nephropathy, or kidney failure, was present. Serum creatinine and eGFR were obtained on the index date or within ±30 days, whereas urinary protein or albumin measurements were selected within ±90 days, according to the temporal selection rules described above.

Cardiac findings were obtained from clinical records, electrocardiography, transthoracic echocardiography, ambulatory rhythm monitoring, and cardiac magnetic resonance imaging when available. Recorded abnormalities included left ventricular hypertrophy, myocardial fibrosis, arrhythmia, conduction disturbance, valvular dysfunction, heart failure, hypertension, and pacemaker or implantable cardioverter-defibrillator implantation. Cardiac involvement was considered present when a structural, functional, or rhythm abnormality attributed to Fabry disease had been documented by the treating cardiologist. Echocardiographic measurements, including interventricular septal thickness, posterior wall thickness, left ventricular mass index, and left ventricular ejection fraction, were recorded when available.

Neurological evaluation included acroparesthesia, chronic neuropathic pain, Fabry pain crises, autonomic symptoms, peripheral neuropathy, electromyography findings, white-matter lesions on brain magnetic resonance imaging, and a history of transient ischemic attack or stroke. Auditory and vestibular manifestations included hearing loss, tinnitus, and vertigo. Hearing loss was recorded when documented by audiometric examination or specialist assessment.

Gastrointestinal involvement included recurrent abdominal pain, diarrhea, constipation, nausea, early satiety, or other chronic gastrointestinal symptoms considered related to Fabry disease. Symptoms were recorded only when documented during routine clinical follow-up.

Dermatological, ocular, and autonomic manifestations included angiokeratomas, cornea verticillata, sweating abnormalities, edema, characteristic facial features, and musculoskeletal complaints. Hepatic involvement, including hepatic steatosis or fibrosis, was recorded when supported by ultrasonography, elastography, computed tomography, magnetic resonance imaging, or a specialist diagnosis.

For the analysis of multiorgan disease, the number of affected non-endocrine organ systems was calculated for each patient. The prespecified domains were renal, cardiac, neurological, gastrointestinal, auditory/vestibular, dermatological/ocular/autonomic, and hepatic involvement. Each affected domain contributed one point to the multiorgan involvement count.

Overall disease burden was assessed using the Mainz Severity Score Index (MSSI) and the Fabry Disease Severity Scoring System (DS3), where sufficient data were available. The MSSI assesses general, neurological, cardiovascular, and renal manifestations [28]. The DS3 evaluates peripheral nervous system, renal, cardiac, central nervous system, and patient-reported domains [29]. For patients with serial assessments, clinical stability or progression was additionally evaluated using the FAbry STabilization indEX (FASTEX, https://www.fastex.online/, accessed on 23 August 2026), which compares changes in neurological, renal, and cardiac domains between consecutive evaluations [30]. Scores were calculated from the documented clinical, laboratory, and imaging findings according to their original definitions. For the cross-sectional analyses, the concurrently performed MSSI and DS3 assessments closest to the patient-specific index date within ±90 days were used. Scores were calculated using clinical information available at the corresponding assessment; when the assessment occurred after the index date, events first documented after the index date were excluded. Because both scores were assessed concurrently, a single temporal interval relative to the index date was reported.

### 2.5. Descriptive Measures and Exploratory Analyses

The present cohort partially overlapped with a previously published cohort from the same center. The previous study specifically evaluated the relationships of serum Lyso-Gb3 concentrations with MSSI, DS3, and FASTEX scores [24]. The present study addressed a different, primarily descriptive question concerning biochemical endocrine findings, vitamin D status, skeletal measurements, and their clinical context. Previously published biomarker–severity relationships were not repeated or presented as novel findings.

Because this was a retrospective study without a prospectively registered statistical analysis plan, no prespecified primary endpoint or confirmatory outcome hierarchy was defined. The elevated-TSH/non-elevated-FT4 biochemical pattern and HbA1c-defined dysglycemia were summarized separately as descriptive biochemical findings. Vitamin D status; available pituitary, adrenal, and gonadal measurements; age-, sex-, and menopausal-status-appropriate DXA findings; and documented organ involvement were also summarized descriptively.

All between-group comparisons and correlation analyses were post hoc and exploratory. For the subgroup comparison, patients with HbA1c-defined dysglycemia (HbA1c ≥ 5.7%; *n* = 7) were compared with patients without HbA1c-defined dysglycemia (HbA1c < 5.7%; *n* = 14). Both patients with an elevated-TSH/non-elevated-FT4 biochemical pattern were already included in the dysglycemia subgroup; therefore, thyroid status did not independently determine subgroup membership. This comparison was consequently treated as a dysglycemia-based stratification rather than as a composite endocrine outcome or a distinct endocrine phenotype. Additional post hoc analyses examined correlations of selected endocrine and skeletal measurements with disease duration, Lyso-Gb3, MSSI, DS3, eGFR, and proteinuria. These analyses were intended solely to generate hypotheses and were not used to establish independent predictors, causal relationships, or excess disease-specific prevalence.

### 2.6. Statistical Analysis

Statistical analyses were performed using IBM SPSS Statistics version 27.0 (IBM Corp., Armonk, NY, USA) and R software version 4.3.0 (R Foundation for Statistical Computing, Vienna, Austria). Continuous variables were assessed for distributional characteristics using the Shapiro–Wilk test together with histograms and quantile–quantile plots. Normally distributed variables were summarized as mean ± standard deviation, whereas non-normally distributed variables were presented as median and interquartile range. Categorical variables were reported as n/N (%). Two-sided exact Clopper–Pearson 95% confidence intervals were calculated for the reported proportions where appropriate. Missing values were not imputed, and all analyses were conducted using available cases, with the relevant denominator reported for each analysis.

All between-group comparisons and correlation analyses were post hoc and exploratory. Patients with HbA1c-defined dysglycemia (HbA1c ≥ 5.7%; *n* = 7) were compared with patients without HbA1c-defined dysglycemia (HbA1c < 5.7%; *n* = 14). For continuous variables, between-group comparisons were performed using the exact permutation form of the Mann–Whitney U test. Rank-biserial correlation coefficients were calculated as effect-size estimates, with positive values indicating higher values in the HbA1c-defined dysglycemia subgroup. Percentile-bootstrap 95% confidence intervals for rank-biserial correlation coefficients were estimated using 20,000 resamples. Categorical variables were compared using Fisher’s exact test, and conditional exact odds ratios with exact 95% confidence intervals were reported.

Exploratory relationships between endocrine or skeletal measurements and Fabry disease-related clinical variables were evaluated using Spearman’s rank correlation coefficient. Confidence intervals for Spearman’s ρ were estimated using 10,000 paired bootstrap resamples. Two-sided *p* values for the correlations were calculated using 50,000 Monte Carlo permutations.

The Benjamini–Hochberg procedure was used to control the false discovery rate within two separately defined exploratory analysis families. The first family comprised the 14 post hoc comparisons between patients with and without HbA1c-defined dysglycemia. The second family comprised the 48 correlations between eight endocrine or skeletal parameters and six Fabry disease-related variables. Benjamini–Hochberg adjustment was performed separately within each family. Both unadjusted *p* values and adjusted q values were reported. An adjusted q value < 0.05 was considered evidence of statistical robustness after multiplicity correction; findings with an unadjusted *p* < 0.05 but q ≥ 0.05 were regarded as nominal and hypothesis-generating only.

Because of the small cohort and limited number of outcome events, no multivariable regression model was fitted. Therefore, the exploratory analyses could not determine whether the observed relationships were independent of age, sex, genotype, disease duration, treatment duration, renal function, menopausal status, or other potential confounders.

## 3. Results

Between 1 January 2020, and 31 December 2025, the records of 29 patients with a documented or suspected diagnosis of Fabry disease were screened. Eight patients were excluded: two were younger than 18 years, one did not have genetically or enzymatically confirmed Fabry disease, one did not have regular institutional follow-up, one had an active infection or acute inflammatory condition at the index assessment, one was pregnant, and two had insufficient or inaccessible core medical records. The remaining 21 patients constituted the final study cohort. Core demographic, clinical, and biochemical endocrine data were available for all 21 patients, whereas organ-involvement assessments and DXA measurements were available for 20 patients. Individual gonadal measurements were available in parameter-specific subsets of the cohort (Figure 1).

Lyso-Gb3 measurements, DXA examinations, and concurrent MSSI/DS3 assessments were available for 21, 20, and 20 patients, respectively. The median absolute intervals from the patient-specific index date were 14 days (range, 0–84 days) for Lyso-Gb3, 63 days (range, 0–176 days) for DXA, and 7 days (range, 0–82 days) for the concurrent MSSI/DS3 assessment. Measurements outside the specified temporal windows were treated as unavailable and were not included in the cross-sectional analyses.

The final cohort comprised 21 patients with a mean age of 40.7 ± 14.5 years (range, 18–72 years); nine (42.9%) were male and hemizygous, and 12 (57.1%) were female and heterozygous. The genetic composition was markedly concentrated in the c.851T>C (p.M284T) variant, which was identified in 12 of 21 patients (57.1%; exact 95% CI, 34.0–78.2%). The remaining patients carried c.605G>C (p.C202S) (3/21, 14.3%), c.376A>G (p.S126G) (3/21, 14.3%), c.782G>A (p.G261D) (2/21, 9.5%), or c.1150A>C (p.I384L) (1/21, 4.8%). Because the remaining nine patients were distributed across four different variants, genotype-stratified inferential analyses were not performed. Consequently, all observed endocrine, skeletal, and organ-specific proportions should be interpreted within the context of this highly selected genetic composition.

All patients were receiving ERT; 15 (71.4%) were treated with agalsidase alfa and six (28.6%) with agalsidase beta. The mean disease and treatment durations were 8.3 ± 4.4 and 6.7 ± 4.3 years, respectively. The median serum Lyso-Gb3 concentration was 4.79 nmol/L (IQR, 2.48–38.47). The demographic, genetic, treatment-related, biochemical, and clinical characteristics are summarized in Table 1.

DXA measurements were available for 20 of the 21 patients. T-score-based interpretation was applicable to eight patients, comprising four postmenopausal women and four men aged ≥50 years. Within this subgroup, five patients (62.5%; exact 95% CI, 24.5–91.5%) had a minimum T-score within the osteoporosis range, two (25.0%) had low bone mass, and one (12.5%) had a T-score ≥ −1.0. Z-score-based interpretation was applicable to 12 patients, comprising seven premenopausal women and five men aged <50 years. Seven of these patients (58.3%; exact 95% CI, 27.7–84.8%) had a minimum Z-score ≤ −2.0, indicating BMD below the expected range for age, whereas five (41.7%) had values within the expected range (Table 2).

Endocrine, metabolic, mineral, and BMD measurements are shown in Table 3. Endocrine, metabolic, and mineral measurements were available for all 21 patients, except for selected gonadal parameters. Estradiol was available in 13 patients (61.9%), total testosterone in 20 (95.2%), inhibin B in nine (42.9%), and AMH in 10 of the 12 female patients (83.3%). The mean serum IGF-1 concentration was 95.2 ± 40.9 ng/mL. The median HbA1c was 5.40% (IQR, 5.20–5.80%), whereas the mean serum 25-hydroxyvitamin D concentration was 15.9 ± 6.4 ng/mL. DXA measurements were available in 20 patients (95.2%). Site-specific T-score summaries were restricted to the T-score-eligible subgroup comprising postmenopausal women and men aged ≥50 years (*n* = 8), whereas site-specific Z-score summaries were restricted to the Z-score-eligible subgroup comprising premenopausal women and men aged <50 years (*n* = 12) (Table 3).

Organ involvement data were available for 20 of the 21 patients. Cardiac and renal involvement were identified in 60.0% and 50.0% of the assessed patients, respectively, whereas central nervous system involvement was present in 15.0%. Nine patients (45.0%) had involvement of at least two major organ systems, and two patients (10.0%) had concurrent renal, cardiac, and central nervous system involvement. Peripheral and autonomic manifestations were frequent, including acroparesthesia in all evaluated patients, sweating abnormalities in 90.0%, and pain crises in 85.0%. Gastrointestinal and auditory/vestibular manifestations were each observed in 75.0% of the evaluated population (Table 4, Figure 2).

Post hoc exploratory analyses compared patients with HbA1c-defined dysglycemia (*n* = 7) with those without HbA1c-defined dysglycemia (*n* = 14) and examined correlations between selected endocrine or skeletal measurements and Fabry disease-related variables. The HbA1c-defined dysglycemia subgroup was substantially older and had longer disease and ERT durations, indicating major potential confounding. None of the 14 subgroup comparisons remained significant after Benjamini–Hochberg correction. Similarly, none of the 48 exploratory correlations remained significant after multiplicity correction. Accordingly, no statistically robust association between the evaluated biochemical endocrine or skeletal measurements and Fabry disease burden was identified, while independent relationships could not be assessed because multivariable adjustment was not feasible. Complete exploratory estimates, confidence intervals, unadjusted *p* values, and adjusted q values are presented in Supplementary Tables S1 and S2.

## 4. Discussion

This small, single-center study descriptively characterized biochemical thyroid and glycemic findings, vitamin D status, age-appropriate DXA measurements, and organ involvement in adults with FD receiving ERT. An elevated-TSH/non-elevated-FT4 biochemical pattern was observed in 9.5% of patients, HbA1c-defined dysglycemia in 33.3%, and vitamin D concentrations < 20 ng/mL in 76.2%. Age-, sex-, and menopausal-status-appropriate DXA interpretation also identified low measurements in subsets of the cohort. However, because the study did not include an age-, sex-, and clinically matched control group, these observed proportions cannot be interpreted as Fabry-specific abnormalities or as being higher than the corresponding frequencies in the general population.

The wide age range of the cohort and the incomplete assessment of conventional metabolic and skeletal risk factors further limit interpretation. Age, menopausal status, body composition, physical activity, dietary intake, sunlight exposure, vitamin D supplementation, renal function, medication use, and other comorbidities may have contributed to the observed biochemical and DXA findings. None of the post hoc subgroup comparisons or exploratory correlations remained significant after correction for multiple testing. Accordingly, these analyses were not used as a basis for mechanistic interpretation or for proposing a distinct endocrine phenotype.

An important feature of the present cohort was the predominance of the c.851T>C (p.M284T) variant, which was present in 57.1% of participants. Fabry disease exhibits substantial genotype- and sex-dependent heterogeneity in residual α-Gal A activity, Lyso-Gb3 concentrations, age at manifestation, and the extent of renal, cardiac, neurological, and other organ involvement [7,22]. Therefore, the observed endocrine, skeletal, and multiorgan findings may have been influenced by the genetic composition of the cohort. The concentration of a single variant may reflect regional referral patterns, founder effects, or family clustering; however, the present study was not designed to distinguish among these possibilities. Because the remaining patients were distributed across four variants with very small cell counts, genotype-specific comparisons would have been statistically unstable and were not performed. The observed proportions should therefore be interpreted as characteristics of this single-center cohort rather than estimates applicable to the genetically heterogeneous Fabry disease population.

This caution is consistent with recent evidence concerning attenuated lysosomal storage disorders. Urizar et al. emphasized that hypomorphic or missense variants permitting residual enzyme activity may be associated with later-onset, subtle, or organ-predominant presentations and that enzyme activity and circulating biomarker concentrations do not invariably predict clinical severity [31]. In Fabry disease, attenuated variants and heterozygosity in women may also be associated with relatively low or non-diagnostic Lyso-Gb3 concentrations despite clinically relevant organ involvement. These considerations further limit genotype–phenotype extrapolation from the present cohort, particularly given the predominance of a single GLA variant and the very small numbers representing the remaining variants.

Thyroid dysfunction is the most consistently reported endocrine abnormality in Fabry disease. Hauser et al. reported subclinical hypothyroidism in four of 11 patients (36.4%), including patients with both preserved and severely impaired renal function [15]. Faggiano et al. subsequently identified antibody-negative subclinical primary hypothyroidism in three of 18 patients (16.7%) and demonstrated higher TSH concentrations in patients with Fabry disease than in matched controls [14]. Bothou et al., in a larger prospective cohort of 77 patients, identified newly diagnosed subclinical hypothyroidism in 3.9%, overt hypothyroidism in 2.6%, and previously known hypothyroidism in 7.8%, producing an overall hypothyroidism prevalence of approximately 14% [16]. The observed proportion of patients with an elevated-TSH/non-elevated-FT4 pattern was 9.5%. Although this value falls within the broad range reported in previous Fabry cohorts, direct comparison is limited by differences in age, renal function, thyroid assessment, treatment exposure, and patient selection. In the absence of a matched control group and systematic thyroid autoantibody and ultrasonographic assessment, the present finding cannot be attributed specifically to FD.

Faggiano et al. proposed that thyroid dysfunction in FD might be related to intrathyroidal glycosphingolipid accumulation and microvascular injury rather than thyroid autoimmunity [14]. A subsequent study reported changes in TSH concentrations and thyroid ultrasonographic findings during ERT [32]. However, the present cross-sectional study did not include pretreatment thyroid assessments, serial thyroid measurements, an untreated comparison group, or systematic thyroid antibody and ultrasonographic data. Therefore, our findings cannot determine whether the observed biochemical thyroid pattern was related to FD, modified by ERT, or attributable to background thyroid disease.

Exploratory analyses did not identify any association between thyroid-related measurements and Fabry disease variables that remained significant after correction for multiple testing; therefore, these findings were not interpreted further.

Seven patients had a single HbA1c value ≥ 5.7%, including five with values in the prediabetes range and two with values in the diabetes range. Bothou et al. reported impaired glucose metabolism in two of 77 patients with FD, whereas Ersoy et al. observed higher HbA1c values in ERT-treated patients with FD than in their Gaucher disease comparison group [16,17]. Hung et al., in a nationwide Taiwanese study of 2352 patients with FD, reported diagnosed diabetes in 35.8% of patients with FD compared with 29.6% of propensity-score-matched controls (adjusted odds ratio, 1.42) [33]. These findings are not directly comparable with ours because the present classification relied on a single HbA1c measurement, included prediabetes-range values, and was not supported by confirmatory testing. The post hoc HbA1c-based subgroup analysis was not interpreted further because no comparison remained significant after multiplicity correction, the groups differed markedly in age and disease duration, and multivariable adjustment was not feasible.

The available pituitary, adrenal, and gonadal measurements did not demonstrate a consistent pattern of clinically established dysfunction. Bothou et al. found no major systematic abnormalities of the adrenal, growth hormone, or sex-hormone axes in their prospective cohort [16]. Maione et al. reported a higher prevalence of empty sella and smaller pituitary volume in patients with Fabry disease than in matched controls, but pituitary function remained substantially preserved [34]. These findings are compatible with the absence of clear GH–IGF-1 abnormalities in our cohort. Nevertheless, basal GH and random cortisol values are not sufficient to exclude growth hormone deficiency or adrenal insufficiency; dynamic testing should remain symptom-driven.

Faggiano et al. reported menstrual or obstetric abnormalities in 89% of women and abnormal semen parameters in all evaluated men [14]. In contrast, Bothou et al. found no biochemical hypogonadism among men and no systematic disruption of FSH, LH, estradiol, AMH, or androgen status among women, apart from a small number of individual reproductive disorders [16]. The present study cannot resolve this discrepancy because estradiol was available in 13 patients, testosterone in 20, inhibin in nine, and AMH in 10 women, while semen analysis, standardized menstrual-cycle timing, and detailed fertility outcomes were unavailable. The gonadal measurements should therefore be regarded as descriptive rather than as evidence for or against Fabry-associated gonadal dysfunction.

A serum 25-hydroxyvitamin D concentration < 20 ng/mL was observed in 16 of 21 patients (76.2%). Bothou et al. reported concentrations below this threshold in 46.8% of 77 patients, while Drechsler et al. reported vitamin D concentrations ≤ 30 ng/mL in approximately 73% of 111 patients [16,35]. However, differences in thresholds, age distribution, geographic location, season of sampling, supplementation, sunlight exposure, renal function, diet, and physical activity limit comparisons across studies. Because the present study lacked a matched control group and did not comprehensively document these conventional determinants, the observed frequency cannot be interpreted as evidence that vitamin D deficiency is specifically associated with FD.

Low vitamin D concentrations may be influenced by numerous factors, including season of sampling, sunlight exposure, dietary intake, supplementation, physical activity, renal function, gastrointestinal disorders, and medication use. These determinants were not comprehensively or uniformly documented in the present retrospective dataset. Although an exploratory correlation between vitamin D concentration and disease duration yielded an unadjusted *p* value < 0.05, it did not remain significant after correction for multiple testing and was not interpreted further. The present data therefore do not support a Fabry-specific mechanism for the observed vitamin D concentrations.

The DXA findings were interpreted according to age, sex, and menopausal status rather than by applying pooled T-score categories to the entire cohort. Among the eight patients for whom T-score-based interpretation was appropriate—postmenopausal women and men aged 50 years or older—five (62.5%) had a minimum T-score within the osteoporosis range, two (25.0%) had low bone mass, and one (12.5%) had a T-score ≥ −1.0. Among the 12 premenopausal women and men younger than 50 years, seven (58.3%) had a minimum Z-score ≤ −2.0, indicating BMD below the expected range for age. Importantly, a Z-score ≤ −2.0 in a younger adult does not independently establish a diagnosis of osteoporosis. These subgroup-specific findings are not clinically interchangeable and should not be combined into a single whole-cohort prevalence estimate.

Previous studies support the presence of reduced BMD in some patients with FD, although reported frequencies have varied considerably. Mersebach et al. identified osteopenia at the hip or lumbar spine in approximately half of 53 untreated patients and found that body weight, renal function, genotype, and menopausal status contributed substantially to BMD variability [18]. More recently, Shmara et al. reported Z-scores ≤ −2.0 in 24% of 25 patients and abnormal T-scores in 86.7% of the subgroup comprising postmenopausal women and men aged ≥50 years [19]. The proportion of younger patients with Z-scores below the expected range for age was numerically higher in our cohort. However, direct comparisons are limited by the small subgroup denominators, wide confidence intervals, different genotype distributions, variable skeletal sites, and differences in conventional osteoporosis risk factors and referral patterns.

Varaldo et al. demonstrated lower lumbar spine BMD and trabecular bone scores in patients with FD than in age-, sex-, and BMI-matched controls, suggesting that skeletal involvement may include impaired bone microarchitecture in addition to reduced areal BMD [36]. Our study did not include a matched control group, trabecular bone score, vertebral fracture assessment, fracture outcomes, or standardized bone-turnover markers. Consequently, the present findings cannot determine whether the observed DXA abnormalities occurred more frequently than expected in the general population or were attributable specifically to FD.

Previous studies have investigated possible relationships between BMD and Fabry-related biochemical or clinical variables. Shmara et al. reported inverse associations of Lyso-Gb3 with skeletal Z- and T-scores but did not identify significant associations with disease duration, ERT duration, renal function, or vitamin D status [19]. In the present cohort, none of the exploratory correlations involving BMD remained significant after Benjamini–Hochberg correction. Given the small sample size, wide confidence intervals, absence of multivariable adjustment, and potential confounding by age, sex, menopausal status, genotype, body composition, renal function, and treatment exposure, these analyses provide no statistically robust evidence that BMD reflects Fabry-specific biochemical activity, cumulative disease exposure, or overall clinical severity.

All participants were receiving ERT; however, the cross-sectional design and absence of pretreatment, untreated, or alternative-treatment comparison groups preclude any assessment of treatment effects on BMD. Aitken et al. reported no significant treatment-group difference in longitudinal lumbar spine Z-score trajectories, while findings at the total hip and femoral neck may have been influenced by baseline disease severity and treatment selection [20]. Our findings neither demonstrate persistent Fabry-specific skeletal involvement despite ERT nor indicate treatment failure or a detrimental treatment effect. Prospective studies incorporating matched controls, pretreatment measurements, conventional skeletal risk factors, fracture outcomes, and longitudinal DXA assessments are required.

Organ-specific manifestations were descriptively documented in the study cohort. Cardiac and renal involvement was recorded in 60.0% and 50.0% of evaluated patients, respectively, while 45.0% had involvement of at least two major organ systems. Peripheral neurological, autonomic, gastrointestinal, and auditory/vestibular manifestations were also frequently recorded. Previous Fabry cohorts have similarly demonstrated involvement across multiple organ systems [37,38]. However, differences in age, genotype, phenotype, treatment exposure, referral patterns, and methods of symptom ascertainment preclude direct comparison of frequencies across studies. The present findings therefore describe the clinical composition of this single-center cohort but do not provide population prevalence estimates.

Recent real-world evidence also highlights the importance of underlying disease chronology and baseline renal status in FD. Mannan et al. reported that older age, higher baseline albuminuria, cardiovascular history, and lower baseline eGFR were associated with incident CKD, faster eGFR decline, or adverse renal outcomes in a national-center cohort [39]. These findings support a cautious, descriptive interpretation of the renal findings in the present small cross-sectional cohort.

Although all participants in the present cohort were receiving ERT, contemporary Fabry-specific treatment also includes the oral pharmacological chaperone migalastat for patients with amenable GLA variants. In a post hoc integrated analysis of 97 adults with amenable variants who received migalastat for up to 8.6 years, Hughes et al. reported 22 Fabry-associated renal, cardiac, or cerebrovascular events in 17 patients; lower baseline eGFR was associated with a higher risk of clinical events [40]. Event rates also varied numerically according to age, sex, phenotype, and previous treatment exposure. These findings illustrate that long-term multiorgan outcomes may be influenced by baseline renal function, genotype, phenotype, age, and treatment history. However, direct comparison with our cohort is not appropriate because all participants in the present study received ERT, treatment allocation was not randomized, the amenability of the identified GLA variants to migalastat was not systematically assessed, and no longitudinal treatment-effect analysis was performed. Our findings therefore cannot be used to compare ERT with migalastat or to determine the effect of either treatment on endocrine, skeletal, renal, or multiorgan outcomes.

Taken together, the present findings are limited to descriptive characterization of a small, selected clinical cohort. The study did not include a matched control group, was not powered for adjusted analyses, and did not identify any association that remained significant after correction for multiple testing. Therefore, the results cannot establish increased Fabry-specific frequencies of endocrine or skeletal abnormalities, independent relationships with disease severity, causal mechanisms, or effects of ERT. Any apparent patterns should be regarded solely as hypotheses for evaluation in appropriately controlled prospective studies.

The principal strength of the study is the concurrent descriptive assessment of biochemical endocrine measurements, glucose metabolism, vitamin D status, age-appropriate DXA findings, Lyso-Gb3, clinical severity scores, and organ involvement within the same cohort. Available-case denominators, effect sizes, confidence intervals, and multiplicity-adjusted results were reported transparently. However, this breadth of characterization does not overcome the limitations imposed by the small sample, lack of controls, incomplete measurements, heterogeneous definitions, and inability to perform multivariable adjustment. Accordingly, the study’s contribution is limited to descriptive clinical characterization and hypothesis generation.

Several limitations substantially restrict the interpretation of the present findings. First, this was a retrospective, single-center study involving only 21 patients, with DXA and organ-involvement data available for 20. The cohort was derived from patients evaluated or regularly followed at a tertiary-care center rather than from a population-based Fabry disease sample. Referral patterns, regular follow-up requirements, genotype and family clustering, and the availability of detailed clinical assessments may therefore have preferentially selected patients with greater disease complexity. In particular, the predominance of the c.851T>C (p.M284T) variant limits the generalizability of the findings to the broader genetically heterogeneous Fabry disease population. Accordingly, the observed proportions should not be interpreted as population prevalence estimates. The study also lacked sufficient information and statistical power to separate the potential effects of genotype from those of sex, age, family clustering, disease duration, and treatment exposure.

Second, the absence of an age-, sex-, and clinically matched control group prevents determination of whether the observed endocrine, metabolic, vitamin D, and skeletal findings occurred more frequently than expected in the general population. The small sample size produced imprecise estimates and wide confidence intervals and precluded reliable multivariable adjustment for age, sex, genotype, menopausal status, disease duration, renal function, and treatment duration. All association analyses were post hoc, no result remained significant after correction for multiple testing, and the absence of multivariable adjustment precluded assessment of independent relationships.

Third, all participants were receiving ERT, preventing comparisons with untreated patients or patients receiving other Fabry-specific therapies and precluding conclusions regarding treatment effects. The cross-sectional assessment also prevented evaluation of temporal or causal relationships. Gonadal measurements were incomplete and were not uniformly timed according to menstrual-cycle phase, while standardized fertility histories and semen analyses were unavailable. Basal cortisol, growth hormone, and related measurements without dynamic testing were insufficient to definitively characterize adrenal or growth hormone function. Dysglycemia was primarily defined using a single HbA1c measurement; therefore, prediabetes- and diabetes-range values represented biochemical findings rather than confirmed clinical diagnoses. Vitamin D supplementation, season of sampling, sunlight exposure, dietary intake, physical activity, fracture history, bone turnover markers, antiepileptic medication exposure, and other conventional skeletal risk factors were not comprehensively documented. Although uniform temporal windows were applied around a patient-specific index date, not all biochemical, densitometric, and disease-severity assessments were performed on the same day. Consequently, residual temporal misalignment between variables cannot be excluded, particularly for DXA measurements, and may have attenuated or otherwise influenced the exploratory associations.

Finally, interpretation of the DXA findings was limited by the broad age range, heterogeneous sex and menopausal composition, and small age-appropriate subgroup denominators. Although T- and Z-score categories were reorganized according to ISCD recommendations, the absence of matched controls and incomplete information regarding fracture history, bone turnover markers, physical activity, nutritional status, vitamin D supplementation, menopausal duration, and medication exposure prevented comprehensive skeletal assessment. Larger prospective, multicenter studies incorporating matched controls, standardized endocrine and skeletal measurements, adequate adjustment for confounding variables, and longitudinal follow-up are required to determine whether the observed findings are specifically related to FD.

## 5. Conclusions

In this small, single-center cohort of 21 adults with Fabry disease receiving ERT, thyroid, glycemic, vitamin D, and age-, sex-, and menopausal-status-appropriate DXA measurements were descriptively characterized. Because the study lacked an appropriately matched control group and no exploratory association remained significant after correction for multiple testing, the findings cannot establish excess endocrine or skeletal involvement attributable to Fabry disease or independent relationships with disease burden. These cohort-specific observations should therefore be considered descriptive and hypothesis-generating.

## Figures and Tables

**Figure 1:**
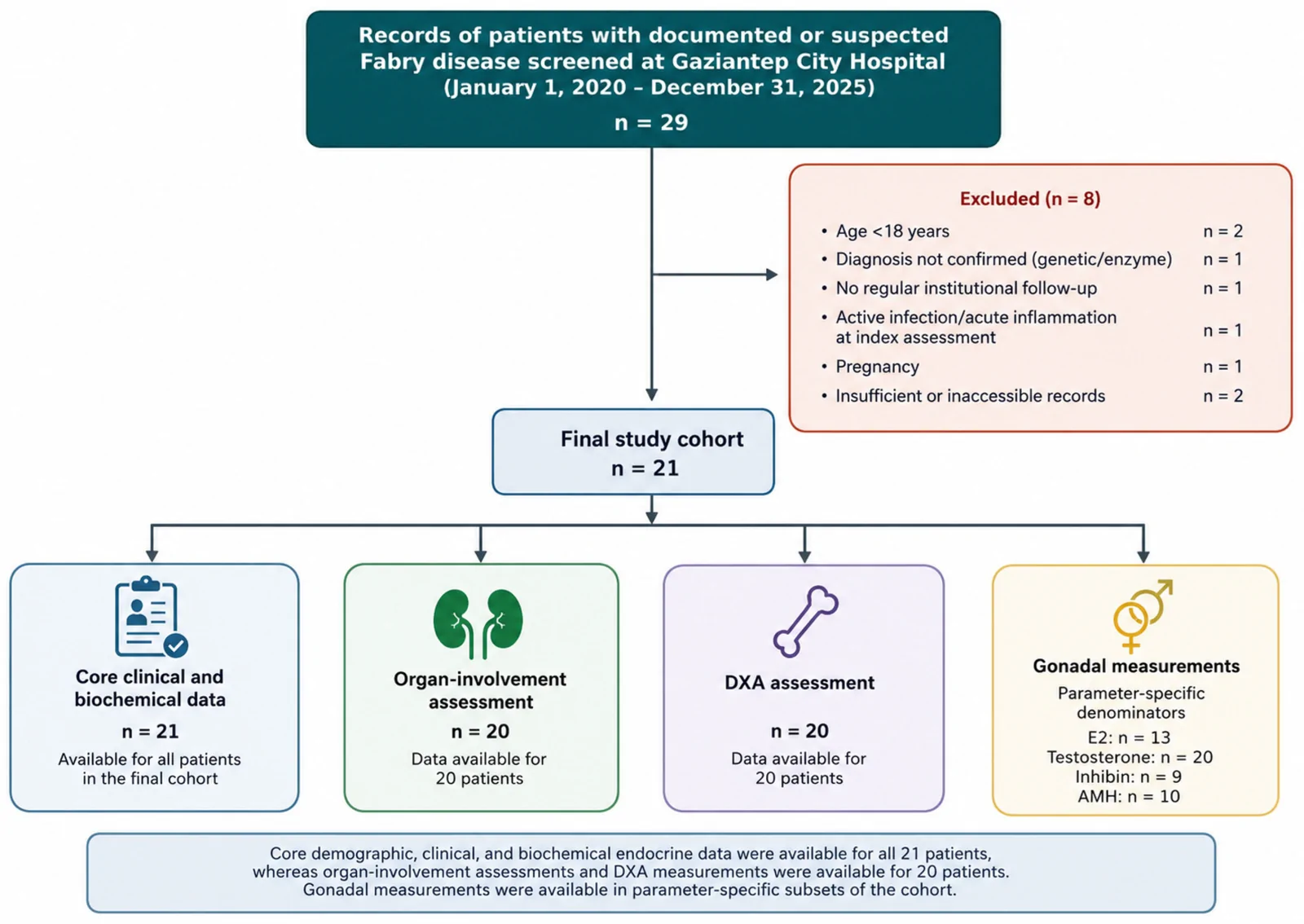
Flow diagram of patient identification, eligibility assessment, exclusions, and formation of the final analytical cohort. DXA, dual-energy X-ray absorptiometry.

**Figure 2:**
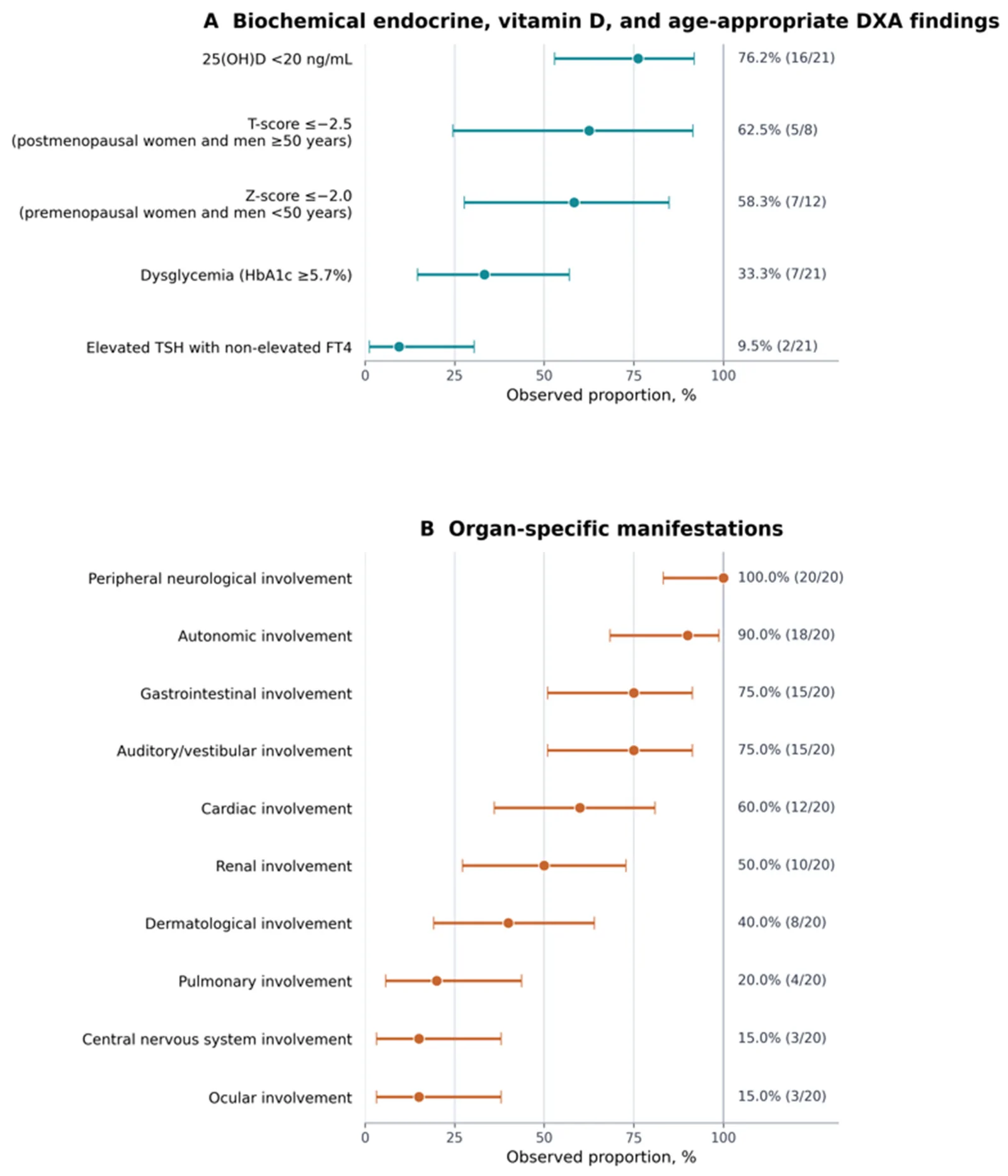
Observed proportions of biochemical endocrine, vitamin D, age-appropriate DXA, and organ-specific findings in the study cohort, with exact 95% confidence intervals. T-score and Z-score estimates were calculated using subgroup-specific denominators according to age, sex, and menopausal status. These observed proportions represent descriptive findings from a single-center cohort and should not be interpreted as population prevalence estimates or as abnormalities attributable specifically to Fabry disease.

**Table 1:** Demographic, genetic, treatment-related, biochemical, and clinical characteristics of the study cohort.

Characteristic	Value, Mean ± SD, Median (IQR), or n (%)
**Demographic and genetic characteristics**	
Age, years	40.7 ± 14.5
Sex	
Male	9 (42.9)
Female	12 (57.1)
Genetic status	
Hemizygous	9 (42.9)
Heterozygous	12 (57.1)
**GLA variant**	
c.851T>C (p.M284T)	12 (57.1)
c.605G>C (p.C202S)	3 (14.3)
c.376A>G (p.S126G)	3 (14.3)
c.782G>A (p.G261D)	2 (9.5)
c.1150A>C (p.I384L)	1 (4.8)
**Disease and treatment characteristics**	
Disease duration, years	8.3 ± 4.4
Receiving enzyme replacement therapy	21 (100.0)
ERT type among treated patients	
Agalsidase alfa	15 (71.4)
Agalsidase beta	6 (28.6)
ERT duration, years	6.7 ± 4.3
**Biochemical and renal characteristics**	
Serum Lyso-Gb3, nmol/L	4.79 (2.48–38.47)
Serum creatinine, mg/dL	0.72 (0.60–0.93)
eGFR, mL/min/1.73 m^2^	104 (95–111)
Serum albumin, g/L	40.5 ± 3.6
Urinary protein excretion, mg/day	126.0 (91.8–275.5)
**Clinical disease-burden scores**	
MSSI total score	26.3 ± 15.3
DS3 total score	18.0 (12.0–22.0)

Data are presented as mean ± standard deviation, median (interquartile range), or n (%), as appropriate. Percentages for ERT type were calculated using all 21 patients receiving ERT as the denominator. Available-case analysis was used for variables with missing data. DS3, Disease Severity Scoring System; eGFR, estimated glomerular filtration rate; ERT, enzyme replacement therapy; Lyso-Gb3, globotriaosylsphingosine; MSSI, Mainz Severity Score Index.

**Table 2:** Age-, sex-, and menopausal-status-appropriate interpretation of DXA findings.

DXA Population and Classification	n/N	%	Exact 95% CI
**DXA measurements available**	20/21	95.2	76.2–99.9
**T-score-eligible subgroup**			
Postmenopausal women and men aged ≥50 years	8/20	40.0	19.1–63.9
T-score ≥ −1.0	1/8	12.5	0.3–52.7
T-score > −2.5 and <−1.0: low bone mass	2/8	25.0	3.2–65.1
T-score ≤ −2.5: osteoporosis range	5/8	62.5	24.5–91.5
**Z-score-eligible subgroup**			
Premenopausal women and men aged <50 years	12/20	60.0	36.1–80.9
Z-score > −2.0: within expected range for age	5/12	41.7	15.2–72.3
Z-score ≤ −2.0: below expected range for age	7/12	58.3	27.7–84.8

Data are presented as n/N (%) with two-sided Clopper–Pearson exact 95% confidence intervals. The T-score-eligible subgroup comprised four postmenopausal women and four men aged ≥50 years. The Z-score-eligible subgroup comprised seven premenopausal women and five men aged <50 years. Patient-level classification was based on the lowest valid score obtained at the lumbar spine, femoral neck, or total hip. T-score categories were applied only to postmenopausal women and men aged ≥50 years. A Z-score ≤ −2.0 indicates BMD below the expected range for age and does not independently establish a diagnosis of osteoporosis [27]. BMD, bone mineral density; CI, confidence interval; DXA, dual-energy X-ray absorptiometry.

**Table 3:** Endocrine, metabolic, mineral, and age-appropriate DXA measurements in patients with Fabry disease.

Parameter	Available n	Value
**Thyroid, pituitary, and adrenal parameters**		
TSH, mIU/L	21	1.60 (1.28–2.64)
Free T4, ng/dL	21	0.79 ± 0.10
Free T3, pg/mL	21	3.33 ± 0.20
Prolactin, ng/mL	21	10.95 ± 3.68
ACTH, pg/mL	21	12.0 (11.0–12.0)
Cortisol, µg/dL	21	8.72 ± 2.48
**Growth hormone axis**		
Growth hormone, ng/mL	21	0.09 (0.04–0.25)
IGF-1, ng/mL	21	95.2 ± 40.9
**Gonadal parameters**		
FSH, men, mIU/mL	9	4.00 (2.41–5.69)
FSH, women, mIU/mL	12	5.99 (4.75–12.25)
LH, men, mIU/mL	9	3.89 ± 1.85
LH, women, mIU/mL	12	5.25 (4.00–13.75)
Estradiol, pg/mL	13	51.1 ± 35.3
Testosterone, men, ng/dL	9	327.2 ± 157.7
Testosterone, women, ng/dL	11	33.3 ± 16.5
Inhibin B, pg/mL	9	20.0 (2.0–181.0)
AMH, women, ng/mL	10	2.42 ± 1.68
**Glucose and lipid metabolism**		
HbA1c, %	21	5.40 (5.20–5.80)
Total cholesterol, mg/dL	21	180.9 ± 45.6
LDL cholesterol, mg/dL	21	106.0 ± 34.2
HDL cholesterol, mg/dL	21	49.2 ± 12.2
Triglycerides, mg/dL	21	112 (88–165)
**Mineral metabolism**		
Calcium, mg/dL	21	9.21 ± 0.54
Phosphorus, mg/dL	21	3.00 (2.78–3.43)
Magnesium, mg/dL	21	2.00 (1.84–2.10)
25-hydroxyvitamin D, ng/mL	21	15.9 ± 6.4
**Bone mineral density measurements**		
Lumbar spine L1–L4 T-score	8	−2.36 ± 0.95
Lumbar spine L1–L4 Z-score	12	−1.88 ± 0.86
Lumbar spine L1–L4 BMD, g/cm^2^	20	0.927 ± 0.140
Femoral neck T-score	8	−2.19 ± 0.75
Femoral neck Z-score	12	−1.74 ± 0.71
Femoral neck BMD, g/cm^2^	20	0.800 ± 0.130
Total hip T-score	8	−1.89 ± 0.79
Total hip Z-score	12	−1.52 ± 0.83
Total hip BMD, g/cm^2^	20	0.889 ± 0.142

Data are presented as mean ± standard deviation or median (interquartile range), according to their distribution. The “Available n” column indicates the number of patients contributing to each estimate. Site-specific T-score summaries were restricted to postmenopausal women and men aged ≥50 years, whereas site-specific Z-score summaries were restricted to premenopausal women and men aged <50 years. Areal BMD was summarized among all patients with a valid measurement at the corresponding skeletal site. Gonadal measurements were analyzed using parameter-specific available cases, and no missing values were imputed. ACTH, adrenocorticotropic hormone; AMH, anti-Müllerian hormone; BMD, bone mineral density; FSH, follicle-stimulating hormone; HDL, high-density lipoprotein; IGF-1, insulin-like growth factor 1; LDL, low-density lipoprotein; LH, luteinizing hormone; TSH, thyroid-stimulating hormone.

**Table 4:** Observed frequencies of organ-specific manifestations and multiorgan involvement in the study cohort.

Organ System/Manifestation	n/N or Median (IQR)	%	Exact 95% CI
**Renal involvement**			
Renal involvement (DS3 renal domain score > 0)	10/20	50.0	27.2–72.8
Proteinuria ≥ 150 mg/24 h	9/20	45.0	23.1–68.5
eGFR < 60 mL/min/1.73 m^2^	2/21	9.5	1.2–30.4
**Cardiac involvement**			
Cardiac involvement (DS3 cardiac domain score > 0)	12/20	60.0	36.1–80.9
Left ventricular hypertrophy	12/20	60.0	36.1–80.9
Arrhythmia	3/20	15.0	3.2–37.9
Heart failure	2/20	10.0	1.2–31.7
**Neurological involvement**			
Central nervous system involvement (DS3 CNS domain score > 0)	3/20	15.0	3.2–37.9
White matter lesions	2/20	10.0	1.2–31.7
Transient ischemic attack or stroke	3/20	15.0	3.2–37.9
Acroparesthesia	20/20	100.0	83.2–100.0
Pain crises	17/20	85.0	62.1–96.8
**Gastrointestinal and autonomic manifestations**			
Gastrointestinal involvement ^a^	15/20	75.0	50.9–91.3
Abdominal pain	15/20	75.0	50.9–91.3
Diarrhea and/or constipation	3/20	15.0	3.2–37.9
Sweating abnormality	18/20	90.0	68.3–98.8
**Other Fabry-related manifestations**			
Angiokeratoma	8/20	40.0	19.1–63.9
Cornea verticillata	3/20	15.0	3.2–37.9
Auditory/vestibular involvement ^b^	15/20	75.0	50.9–91.3
Tinnitus	8/20	40.0	19.1–63.9
Vertigo	15/20	75.0	50.9–91.3
Pulmonary involvement	4/20	20.0	5.7–43.7
**Major-organ burden ^c^**			
Number of major organ systems involved	1 (0–2)	—	—
Multiorgan involvement (≥2 major organ systems)	9/20	45.0	23.1–68.5
Involvement of all three major organ systems	2/20	10.0	1.2–31.7

Data are presented as n/N (%) or median (interquartile range). Confidence intervals were calculated using the exact Clopper–Pearson method. A recorded MSSI or DS3 component score greater than zero was considered evidence of the corresponding manifestation. ^a^ Gastrointestinal involvement was defined as the presence of abdominal pain and/or diarrhea/constipation. ^b^ Auditory/vestibular involvement was defined as the presence of tinnitus and/or vertigo. ^c^ Major organ systems comprised the renal, cardiac, and central nervous system domains. Multiorgan involvement was defined as involvement of at least two of these three domains.

## Data Availability

Data is contained within the article or Supplementary Materials.

## Supplementary Materials

The following supporting information can be downloaded at: https://www.mdpi.com/article/10.3390/biom16101395/s1, Table S1: Post hoc exploratory comparisons according to HbA1c-defined dysglycemia status. Table S2: Spearman correlation analysis between endocrine and bone health parameters and Fabry disease-related clinical variables.

## Abbreviations

The following abbreviations are used in this manuscript:

ACTH	Adrenocorticotropic hormone	GLA	Alpha-galactosidase A gene
α-Gal A	Alpha-galactosidase A	HbA1c	Glycated hemoglobin
AMH	Anti-Müllerian hormone	HDL-C	High-density lipoprotein cholesterol
BH	Benjamini–Hochberg	HOMA-IR	Homeostatic Model Assessment of Insulin Resistance
BMD	Bone mineral density	IGF-1	Insulin-like growth factor 1
CI	Confidence interval	IQR	Interquartile range
CKD	Chronic kidney disease	LDL-C	Low-density lipoprotein cholesterol
CNS	Central nervous system	LH	Luteinizing hormone
DS3	Disease Severity Scoring System	LV	Left ventricular
DXA	Dual-energy X-ray absorptiometry	LVH	Left ventricular hypertrophy
eGFR	Estimated glomerular filtration rate	Lyso-Gb3	Globotriaosylsphingosine
ERT	Enzyme replacement therapy	MRI	Magnetic resonance imaging
FASTEX	FAbry STabilization indEX	MSSI	Mainz Severity Score Index
FD	Fabry disease	OR	Odds ratio
FSH	Follicle-stimulating hormone	PTH	Parathyroid hormone
FT3	Free triiodothyronine	SD	Standard deviation
FT4	Free thyroxine	TBS	Trabecular bone score
Gb3	Globotriaosylceramide	TSH	Thyroid-stimulating hormone
GH	Growth hormone	25(OH)D	25-Hydroxyvitamin D
GI	Gastrointestinal		
