# Supplementary material for: Biochemical Endocrine and Skeletal Findings in Adults with Fabry Disease: A Descriptive Single-Center Study
Source: Biomolecules. 2026 Sep 24;16(10):1395. doi: 10.3390/biom16101395 (PMC13702526; doi:10.3390/biom16101395)
Supplement: Supplementary file 1 [file biomolecules-16-01395-s001.zip › biomolecules-4572323-supplementary.pdf]

Supplementary Table S1. Post hoc exploratory comparisons according to HbA1c-defined dysglycemia status.

Characteristic	HbA1c-defined dysglycemia (HbA1c ≥5.7%; n = 7)	No HbA1c-defined dysglycemia (HbA1c <5.7%; n = 14)	Effect size (95% CI)	BH-adjusted q value
**Demographic and treatment-related characteristics**				
Age, years	55.0 (48.5–58.0)	32.5 (27.0–42.8)	$r_{r\beta}$=0.73 (0.33–1.00)	0.056
Male sex	4/7 (57.1)	5/14 (35.7)	OR=2.30 (0.27–22.92)	0.428
Disease duration, years	12.0 (9.5–15.0)	6.0 (5.3–9.0)	$r_{r\beta}$=0.56 (0.03–0.95)	0.075
ERT duration, years	10.0 (8.5–14.0)	4.5 (2.5–8.0)	$r_{r\beta}$=0.64 (0.09–1.00)	0.056
Agalsidase beta treatment	1/7 (14.3)	5/14 (35.7)	OR=0.32 (0.01–4.00)	0.613
**Biochemical and clinical severity characteristics**				
Lyso-Gb3	53.97 (6.77–74.27)	3.98 (2.08–27.91)	$r_{r\beta}$=0.65 (0.20–0.96)	0.056
eGFR, mL/min/1.73 m²	96.0 (74.0–99.0)	110.0 (104.3–121.5)	$r_{r\beta}$=−0.68 (−0.98 to −0.29)	0.056
Proteinuria, mg/24 h	180.0 (143.5–699.0)	109.0 (85.0–227.0)	$r_{r\beta}$=0.55 (0.08–0.90)	0.084
MSSI score	32.0 (28.0–46.0)	17.0 (13.0–29.0)	$r_{r\beta}$=0.59 (0.11–0.96)	0.073
DS3 score	22.0 (19.0–37.0)	15.0 (11.0–20.0)	$r_{r\beta}$=0.59 (0.14–0.91)	0.073
**Organ-specific involvement**				
Renal involvement	6/7 (85.7)	4/13 (30.8)	OR=11.64 (0.95–687.88)	0.089
Cardiac involvement	6/7 (85.7)	6/13 (46.2)	OR=6.36 (0.53–366.99)	0.203
Central nervous system involvement	2/7 (28.6)	1/13 (7.7)	OR=4.40 (0.19–306.19)	0.315
Multiorgan involvement (≥2 major systems)	5/7 (71.4)	4/13 (30.8)	OR=5.11 (0.54–76.45)	0.203

Continuous variables are presented as median (interquartile range), and categorical variables as n/N (%). Subgroups were defined solely according to HbA1c-defined dysglycemia (HbA1c ≥5.7% vs. <5.7%); thyroid status did not determine subgroup membership. Continuous variables were compared using the exact permutation form of the Mann–Whitney U test. Effect sizes are presented as rank-biserial correlations with percentile-bootstrap 95% confidence intervals based on 20,000 resamples. Categorical variables were compared using Fisher's exact test, and conditional exact odds ratios with exact 95% confidence intervals are reported. Benjamini–Hochberg q values were calculated across the family of 14 comparisons.

Supplementary Table S2. Spearman correlation analysis between endocrine and bone health parameters and Fabry disease-related clinical variables.

Parameter	Disease duration	Lyso-Gb3	MSSI score	DS3 score	eGFR	Proteinuria
HbA1c	0.31 [−0.18, 0.68]; 0.174	0.32 [−0.14, 0.68]; 0.156	0.41 [−0.03, 0.73]; 0.074	0.36 [−0.07, 0.72]; 0.116	−0.55 [−0.77, −0.19]; 0.011	0.45 [−0.04, 0.82]; 0.047
TSH	0.25 [−0.22, 0.63]; 0.274	0.46 [−0.06, 0.78]; 0.038	0.35 [−0.19, 0.76]; 0.129	0.25 [−0.27, 0.66]; 0.287	−0.04 [−0.50, 0.47]; 0.872	0.36 [−0.14, 0.75]; 0.116
FT4	0.17 [−0.31, 0.62]; 0.458	0.23 [−0.27, 0.67]; 0.306	0.01 [−0.47, 0.53]; 0.979	0.07 [−0.42, 0.59]; 0.751	0.06 [−0.45, 0.50]; 0.808	0.06 [−0.47, 0.56]; 0.790
IGF-1	0.07 [−0.45, 0.58]; 0.748	0.20 [−0.30, 0.61]; 0.375	0.12 [−0.38, 0.57]; 0.612	0.20 [−0.29, 0.64]; 0.387	−0.24 [−0.62, 0.22]; 0.305	0.07 [−0.50, 0.60]; 0.762
25(OH)D	0.49 [0.06, 0.78]; 0.026	0.23 [−0.22, 0.66]; 0.319	0.29 [−0.19, 0.65]; 0.213	0.33 [−0.16, 0.75]; 0.148	−0.39 [−0.70, 0.02]; 0.079	−0.10 [−0.56, 0.38]; 0.661
Lumbar spine L1–L4 BMD	−0.22 [−0.67, 0.33]; 0.348	0.03 [−0.48, 0.52]; 0.897	−0.14 [−0.58, 0.37]; 0.565	−0.04 [−0.51, 0.47]; 0.872	−0.01 [−0.43, 0.41]; 0.975	−0.18 [−0.61, 0.31]; 0.452
Femoral neck BMD	−0.42 [−0.73, 0.02]; 0.067	−0.51 [−0.82, −0.04]; 0.022	−0.53 [−0.83, −0.08]; 0.020	−0.43 [−0.82, 0.11]; 0.066	0.24 [−0.24, 0.63]; 0.309	−0.19 [−0.63, 0.33]; 0.433
Total hip BMD	−0.48 [−0.81, −0.04]; 0.032	−0.36 [−0.74, 0.13]; 0.117	−0.37 [−0.74, 0.10]; 0.120	−0.28 [−0.70, 0.24]; 0.250	0.18 [−0.24, 0.55]; 0.452	−0.04 [−0.51, 0.47]; 0.875

Each cell presents Spearman's ρ with its paired-bootstrap 95% confidence interval and two-sided permutation p value. Confidence intervals were estimated using 10,000 paired bootstrap resamples, and p values were calculated using 50,000 Monte Carlo permutations. The Benjamini–Hochberg procedure was applied across the complete family of 48 correlations. Both unadjusted p values and adjusted q values are provided. No correlation remained statistically significant after false-discovery-rate correction. BMD, bone mineral density; DS3, Disease Severity Scoring System; eGFR, estimated glomerular filtration rate; FT4, free thyroxine; IGF-1, insulin-like growth factor 1; MSSI, Mainz Severity Score Index; 25(OH)D, 25-hydroxyvitamin D.
